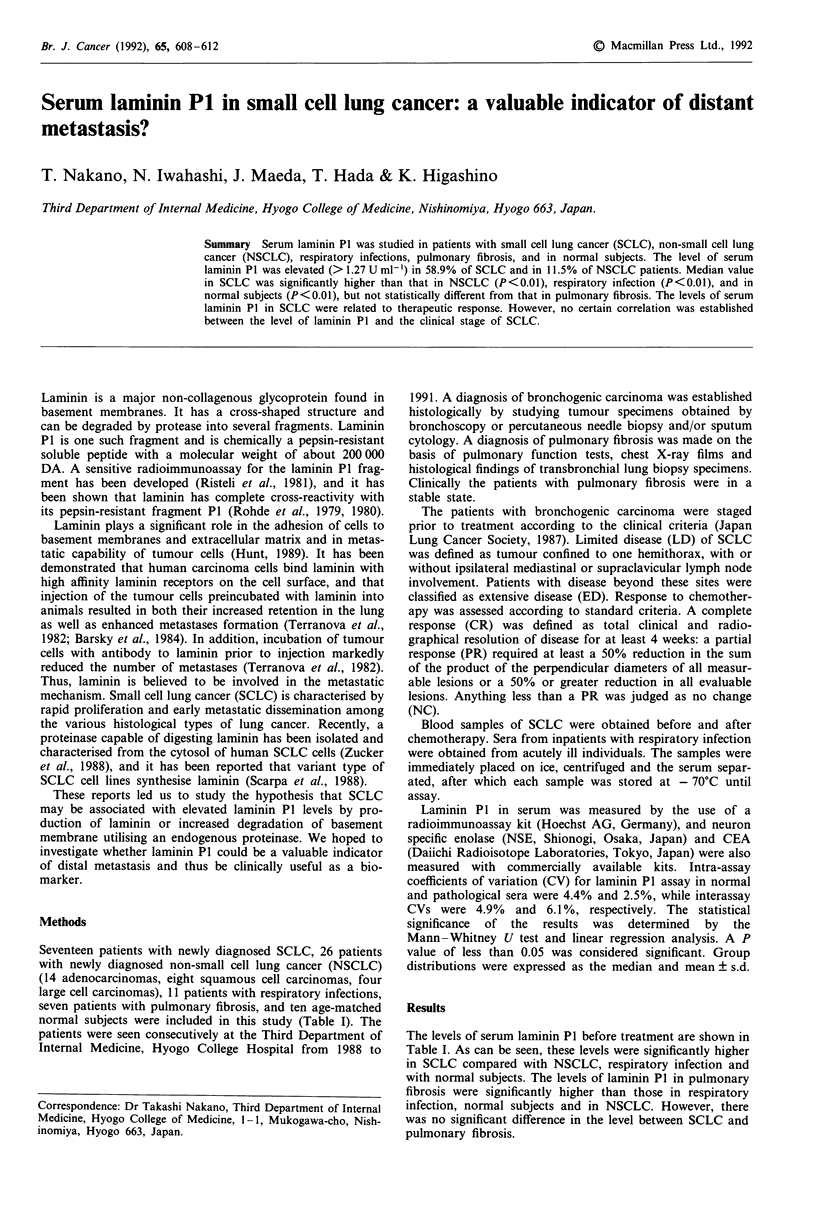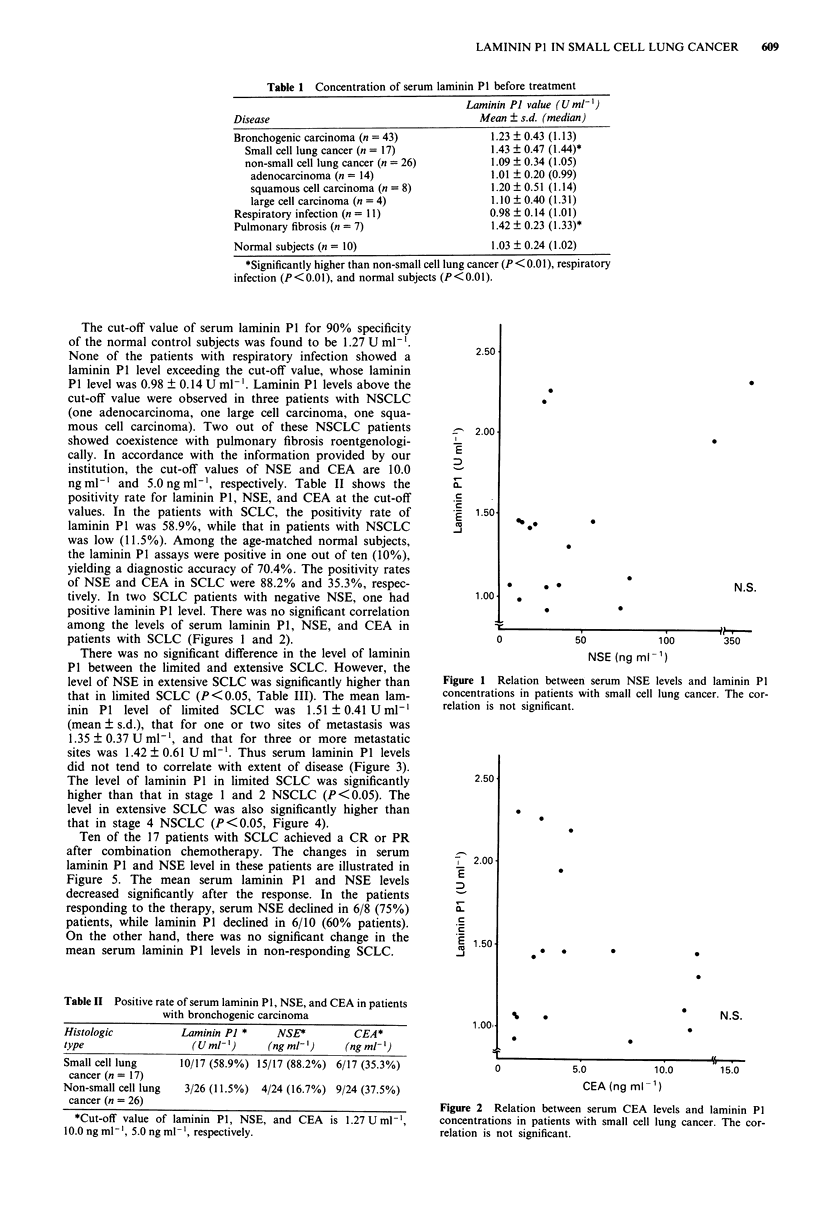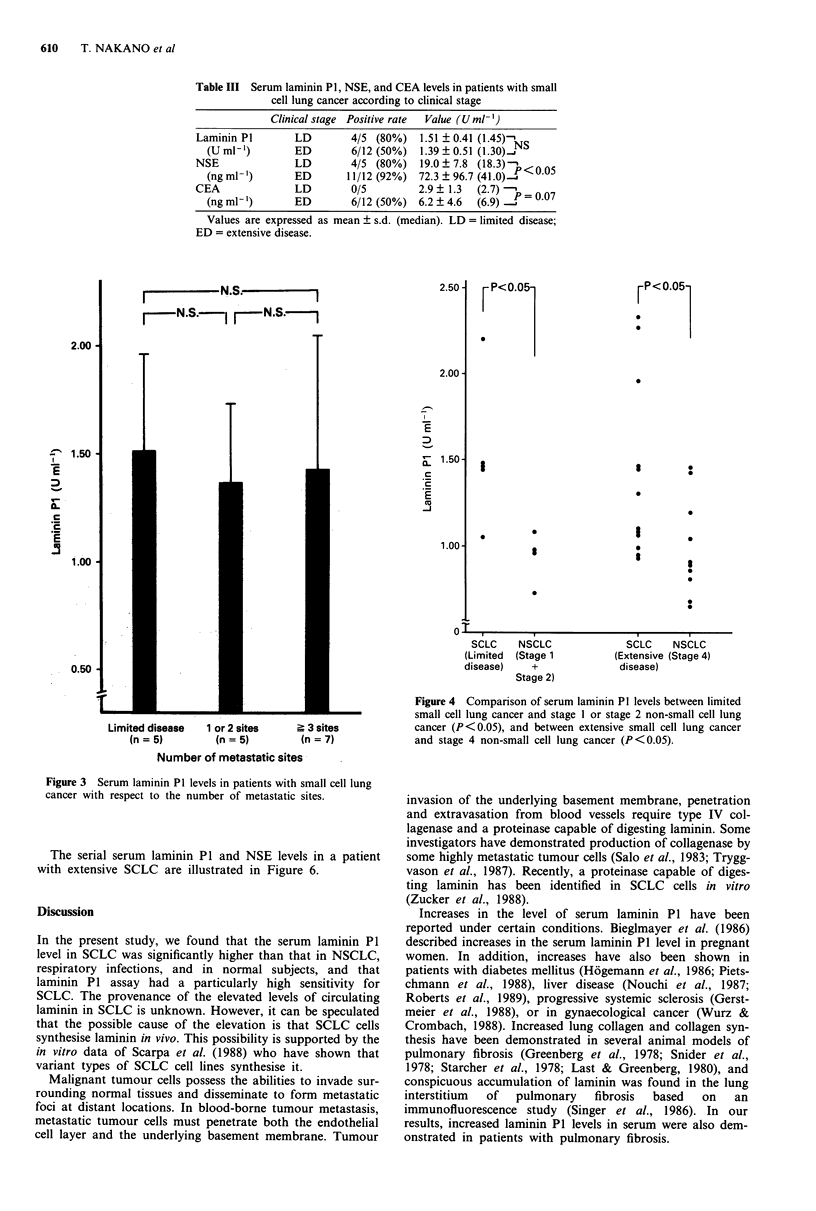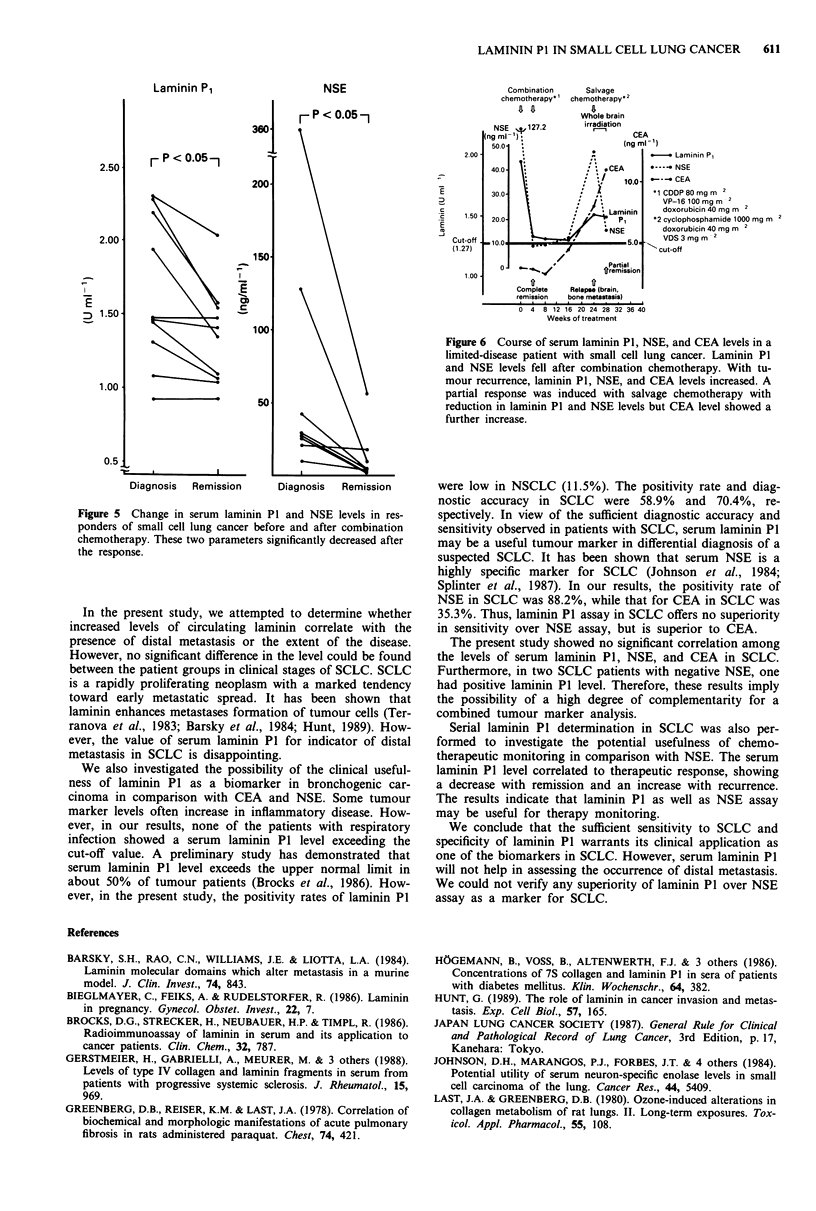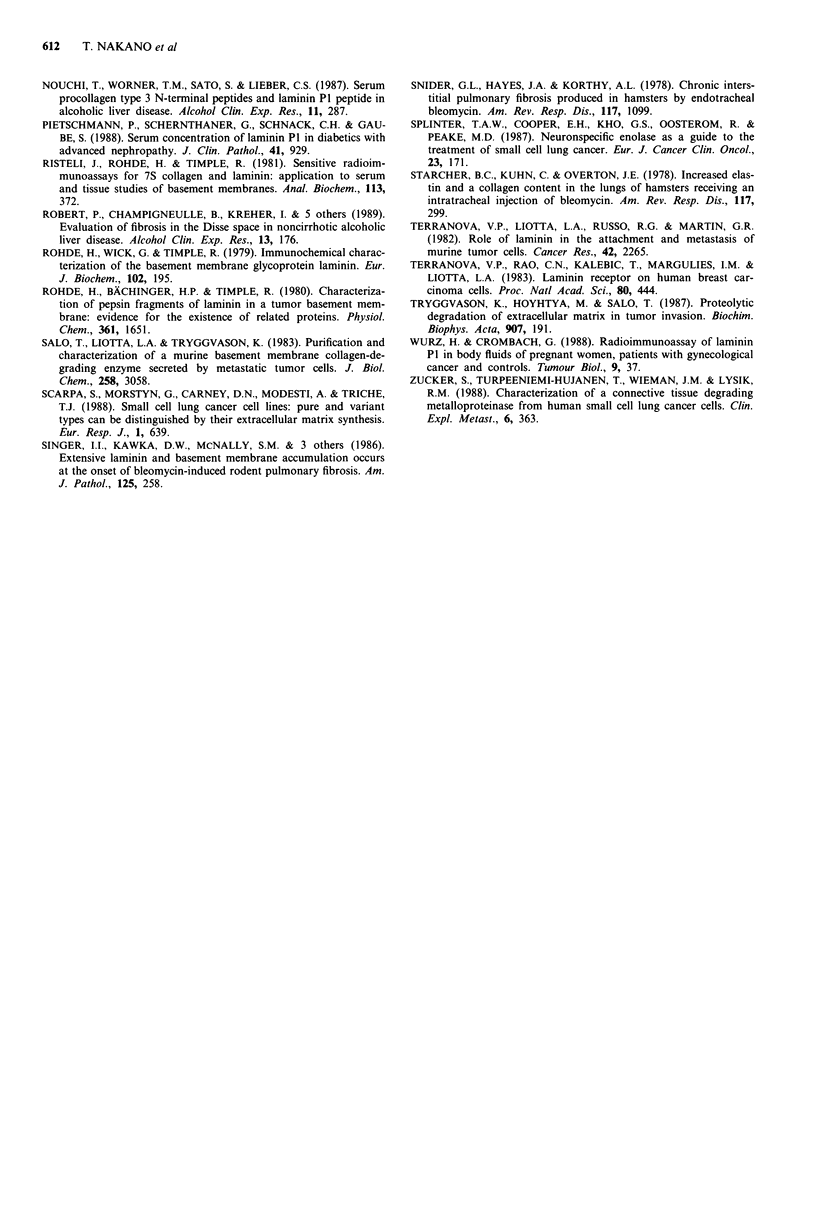# Serum laminin P1 in small cell lung cancer: a valuable indicator of distant metastasis?

**DOI:** 10.1038/bjc.1992.123

**Published:** 1992-04

**Authors:** T. Nakano, N. Iwahashi, J. Maeda, T. Hada, K. Higashino

**Affiliations:** Third Department of Internal Medicine, Hyogo College of Medicine, Japan.

## Abstract

Serum laminin P1 was studied in patients with small cell lung cancer (SCLC), non-small cell lung cancer (NSCLC), respiratory infections, pulmonary fibrosis, and in normal subjects. The level of serum laminin P1 was elevated (greater than 1.27 U ml-1) in 58.9% of SCLC and in 11.5% of NSCLC patients. Median value in SCLC was significantly higher than that in NSCLC (P less than 0.01), respiratory infection (P less than 0.01), and in normal subjects (P less than 0.01), but not statistically different from that in pulmonary fibrosis. The levels of serum laminin P1 in SCLC were related to therapeutic response. However, no certain correlation was established between the level of laminin P1 and the clinical stage of SCLC.


Br. J. Cancer (1992), 65, 608–612 © Macmillan Press Ltd., 1992

### Serum laminin P1 in small cell lung cancer: a valuable indicator of distant metastasis?

T. Nakano, N. Iwahashi, J. Maeda, T. Hada & K. Higashino

*Third Department of Internal Medicine, Hyogo College of Medicine, Nishinomiya, Hyogo 663, Japan.*

**Summary** Serum laminin P1 was studied in patients with small cell lung cancer (SCLC), non-small cell lung cancer (NSCLC), respiratory infections, pulmonary fibrosis, and in normal subjects. The level of serum laminin P1 was elevated (> 1.27 U $ml^{-1}$) in 58.9% of SCLC and in 11.5% of NSCLC patients. Median value in SCLC was significantly higher than that in NSCLC ($P$<0.01), respiratory infection ($P$<0.01), and in normal subjects ($P$<0.01), but not statistically different from that in pulmonary fibrosis. The levels of serum laminin P1 in SCLC were related to therapeutic response. However, no certain correlation was established between the level of laminin P1 and the clinical stage of SCLC.

Laminin is a major non-collagenous glycoprotein found in basement membranes. It has a cross-shaped structure and can be degraded by protease into several fragments. Laminin P1 is one such fragment and is chemically a pepsin-resistant soluble peptide with a molecular weight of about 200 000 DA. A sensitive radioimmunoassay for the laminin P1 fragment has been developed (Risteli *et al.*, 1981), and it has been shown that laminin has complete cross-reactivity with its pepsin-resistant fragment P1 (Rohde *et al.*, 1979, 1980).

Laminin plays a significant role in the adhesion of cells to basement membranes and extracellular matrix and in metastatic capability of tumour cells (Hunt, 1989). It has been demonstrated that human carcinoma cells bind laminin with high affinity laminin receptors on the cell surface, and that injection of the tumour cells preincubated with laminin into animals resulted in both their increased retention in the lung as well as enhanced metastases formation (Terranova *et al.*, 1982; Barsky *et al.*, 1984). In addition, incubation of tumour cells with antibody to laminin prior to injection markedly reduced the number of metastases (Terranova *et al.*, 1982). Thus, laminin is believed to be involved in the metastatic mechanism. Small cell lung cancer (SCLC) is characterised by rapid proliferation and early metastatic dissemination among the various histological types of lung cancer. Recently, a proteinase capable of digesting laminin has been isolated and characterised from the cytosol of human SCLC cells (Zucker *et al.*, 1988), and it has been reported that variant type of SCLC cell lines synthesise laminin (Scarpa *et al.*, 1988).

These reports led us to study the hypothesis that SCLC may be associated with elevated laminin P1 levels by production of laminin or increased degradation of basement membrane utilising an endogenous proteinase. We hoped to investigate whether laminin P1 could be a valuable indicator of distal metastasis and thus be clinically useful as a biomarker.

### Methods

Seventeen patients with newly diagnosed SCLC, 26 patients with newly diagnosed non-small cell lung cancer (NSCLC) (14 adenocarcinomas, eight squamous cell carcinomas, four large cell carcinomas), 11 patients with respiratory infections, seven patients with pulmonary fibrosis, and ten age-matched normal subjects were included in this study (Table I). The patients were seen consecutively at the Third Department of Internal Medicine, Hyogo College Hospital from 1988 to 1991. A diagnosis of bronchogenic carcinoma was established histologically by studying tumour specimens obtained by bronchoscopy or percutaneous needle biopsy and/or sputum cytology. A diagnosis of pulmonary fibrosis was made on the basis of pulmonary function tests, chest X-ray films and histological findings of transbronchial lung biopsy specimens. Clinically the patients with pulmonary fibrosis were in a stable state.

The patients with bronchogenic carcinoma were staged prior to treatment according to the clinical criteria (Japan Lung Cancer Society, 1987). Limited disease (LD) of SCLC was defined as tumour confined to one hemithorax, with or without ipsilateral mediastinal or supraclavicular lymph node involvement. Patients with disease beyond these sites were classified as extensive disease (ED). Response to chemotherapy was assessed according to standard criteria. A complete response (CR) was defined as total clinical and radiographical resolution of disease for at least 4 weeks: a partial response (PR) required at least a 50% reduction in the sum of the product of the perpendicular diameters of all measurable lesions or a 50% or greater reduction in all evaluable lesions. Anything less than a PR was judged as no change (NC).

Blood samples of SCLC were obtained before and after chemotherapy. Sera from inpatients with respiratory infection were obtained from acutely ill individuals. The samples were immediately placed on ice, centrifuged and the serum separated, after which each sample was stored at − 70°C until assay.

Laminin P1 in serum was measured by the use of a radioimmunoassay kit (Hoechst AG, Germany), and neuron specific enolase (NSE, Shionogi, Osaka, Japan) and CEA (Daiichi Radioisotope Laboratories, Tokyo, Japan) were also measured with commercially available kits. Intra-assay coefficients of variation (CV) for laminin P1 assay in normal and pathological sera were 4.4% and 2.5%, while interassay CVs were 4.9% and 6.1%, respectively. The statistical significance of the results was determined by the Mann–Whitney $U$ test and linear regression analysis. A $P$ value of less than 0.05 was considered significant. Group distributions were expressed as the median and mean ± s.d.

### Results

The levels of serum laminin P1 before treatment are shown in Table I. As can be seen, these levels were significantly higher in SCLC compared with NSCLC, respiratory infection and with normal subjects. The levels of laminin P1 in pulmonary fibrosis were significantly higher than those in respiratory infection, normal subjects and in NSCLC. However, there was no significant difference in the level between SCLC and pulmonary fibrosis.

Correspondence: Dr Takashi Nakano, Third Department of Internal Medicine, Hyogo College of Medicine, 1–1, Mukogawa-cho, Nishinomiya, Hyogo 663, Japan.

**Table 1** Concentration of serum laminin P1 before treatment

| Disease | *Laminin P1 value ($U\ ml^{-1}$) Mean ± s.d. (median)* |
|---|---|
| Bronchogenic carcinoma ($n = 43$) | 1.23 ± 0.43 (1.13) |
| Small cell lung cancer ($n = 17$) | 1.43 ± 0.47 (1.44)* |
| non-small cell lung cancer ($n = 26$) | 1.09 ± 0.34 (1.05) |
| adenocarcinoma ($n = 14$) | 1.01 ± 0.20 (0.99) |
| squamous cell carcinoma ($n = 8$) | 1.20 ± 0.51 (1.14) |
| large cell carcinoma ($n = 4$) | 1.10 ± 0.40 (1.31) |
| Respiratory infection ($n = 11$) | 0.98 ± 0.14 (1.01) |
| Pulmonary fibrosis ($n = 7$) | 1.42 ± 0.23 (1.33)* |
| Normal subjects ($n = 10$) | 1.03 ± 0.24 (1.02) |

*Significantly higher than non-small cell lung cancer ($P < 0.01$), respiratory infection ($P < 0.01$), and normal subjects ($P < 0.01$).

The cut-off value of serum laminin P1 for 90% specificity of the normal control subjects was found to be 1.27 $U\ ml^{-1}$. None of the patients with respiratory infection showed a laminin P1 level exceeding the cut-off value, whose laminin P1 level was 0.98 ± 0.14 $U\ ml^{-1}$. Laminin P1 levels above the cut-off value were observed in three patients with NSCLC (one adenocarcinoma, one large cell carcinoma, one squamous cell carcinoma). Two out of these NSCLC patients showed coexistence with pulmonary fibrosis roentgenologically. In accordance with the information provided by our institution, the cut-off values of NSE and CEA are 10.0 $ng\ ml^{-1}$ and 5.0 $ng\ ml^{-1}$, respectively. Table II shows the positivity rate for laminin P1, NSE, and CEA at the cut-off values. In the patients with SCLC, the positivity rate of laminin P1 was 58.9%, while that in patients with NSCLC was low (11.5%). Among the age-matched normal subjects, the laminin P1 assays were positive in one out of ten (10%), yielding a diagnostic accuracy of 70.4%. The positivity rates of NSE and CEA in SCLC were 88.2% and 35.3%, respectively. In two SCLC patients with negative NSE, one had positive laminin P1 level. There was no significant correlation among the levels of serum laminin P1, NSE, and CEA in patients with SCLC (Figures 1 and 2).

There was no significant difference in the level of laminin P1 between the limited and extensive SCLC. However, the level of NSE in extensive SCLC was significantly higher than that in limited SCLC ($P < 0.05$, Table III). The mean laminin P1 level of limited SCLC was 1.51 ± 0.41 $U\ ml^{-1}$ (mean ± s.d.), that for one or two sites of metastasis was 1.35 ± 0.37 $U\ ml^{-1}$, and that for three or more metastatic sites was 1.42 ± 0.61 $U\ ml^{-1}$. Thus serum laminin P1 levels did not tend to correlate with extent of disease (Figure 3). The level of laminin P1 in limited SCLC was significantly higher than that in stage 1 and 2 NSCLC ($P < 0.05$). The level in extensive SCLC was also significantly higher than that in stage 4 NSCLC ($P < 0.05$, Figure 4).

Ten of the 17 patients with SCLC achieved a CR or PR after combination chemotherapy. The changes in serum laminin P1 and NSE level in these patients are illustrated in Figure 5. The mean serum laminin P1 and NSE levels decreased significantly after the response. In the patients responding to the therapy, serum NSE declined in 6/8 (75%) patients, while laminin P1 declined in 6/10 (60% patients). On the other hand, there was no significant change in the mean serum laminin P1 levels in non-responding SCLC.

**Table II** Positive rate of serum laminin P1, NSE, and CEA in patients with bronchogenic carcinoma

| *Histologic type* | *Laminin P1 * ($U\ ml^{-1}$)* | *NSE* ($ng\ ml^{-1}$)* | *CEA* ($ng\ ml^{-1}$)* |
|---|---|---|---|
| Small cell lung cancer ($n = 17$) | 10/17 (58.9%) | 15/17 (88.2%) | 6/17 (35.3%) |
| Non-small cell lung cancer ($n = 26$) | 3/26 (11.5%) | 4/24 (16.7%) | 9/24 (37.5%) |

*Cut-off value of laminin P1, NSE, and CEA is 1.27 $U\ ml^{-1}$, 10.0 $ng\ ml^{-1}$, 5.0 $ng\ ml^{-1}$, respectively.

**Figure 1** Relation between serum NSE levels and laminin P1 concentrations in patients with small cell lung cancer. The correlation is not significant.

**Figure 2** Relation between serum CEA levels and laminin P1 concentrations in patients with small cell lung cancer. The correlation is not significant.

**Table III** Serum laminin P1, NSE, and CEA levels in patients with small cell lung cancer according to clinical stage

| | *Clinical stage* | *Positive rate* | *Value ($U ml^{-1}$)* |
|---|---|---|---|
| Laminin P1 | LD | 4/5 (80%) | 1.51 ± 0.41 (1.45) NS |
| ($U ml^{-1}$) | ED | 6/12 (50%) | 1.39 ± 0.51 (1.30) |
| NSE | LD | 4/5 (80%) | 19.0 ± 7.8 (18.3) $P<0.05$ |
| ($ng ml^{-1}$) | ED | 11/12 (92%) | 72.3 ± 96.7 (41.0) |
| CEA | LD | 0/5 | 2.9 ± 1.3 (2.7) $P=0.07$ |
| ($ng ml^{-1}$) | ED | 6/12 (50%) | 6.2 ± 4.6 (6.9) |

Values are expressed as mean ± s.d. (median). LD = limited disease; ED = extensive disease.

**Figure 3** Serum laminin P1 levels in patients with small cell lung cancer with respect to the number of metastatic sites.

**Figure 4** Comparison of serum laminin P1 levels between limited small cell lung cancer and stage 1 or stage 2 non-small cell lung cancer ($P<0.05$), and between extensive small cell lung cancer and stage 4 non-small cell lung cancer ($P<0.05$).

The serial serum laminin P1 and NSE levels in a patient with extensive SCLC are illustrated in Figure 6.

### Discussion

In the present study, we found that the serum laminin P1 level in SCLC was significantly higher than that in NSCLC, respiratory infections, and in normal subjects, and that laminin P1 assay had a particularly high sensitivity for SCLC. The provenance of the elevated levels of circulating laminin in SCLC is unknown. However, it can be speculated that the possible cause of the elevation is that SCLC cells synthesise laminin *in vivo*. This possibility is supported by the *in vitro* data of Scarpa *et al.* (1988) who have shown that variant types of SCLC cell lines synthesise it.

Malignant tumour cells possess the abilities to invade surrounding normal tissues and disseminate to form metastatic foci at distant locations. In blood-borne tumour metastasis, metastatic tumour cells must penetrate both the endothelial cell layer and the underlying basement membrane. Tumour invasion of the underlying basement membrane, penetration and extravasation from blood vessels require type IV collagenase and a proteinase capable of digesting laminin. Some investigators have demonstrated production of collagenase by some highly metastatic tumour cells (Salo *et al.*, 1983; Tryggvason *et al.*, 1987). Recently, a proteinase capable of digesting laminin has been identified in SCLC cells *in vitro* (Zucker *et al.*, 1988).

Increases in the level of serum laminin P1 have been reported under certain conditions. Bieglmayer *et al.* (1986) described increases in the serum laminin P1 level in pregnant women. In addition, increases have also been shown in patients with diabetes mellitus (Högemann *et al.*, 1986; Pietschmann *et al.*, 1988), liver disease (Nouchi *et al.*, 1987; Roberts *et al.*, 1989), progressive systemic sclerosis (Gerstmeier *et al.*, 1988), or in gynaecological cancer (Wurz & Crombach, 1988). Increased lung collagen and collagen synthesis have been demonstrated in several animal models of pulmonary fibrosis (Greenberg *et al.*, 1978; Snider *et al.*, 1978; Starcher *et al.*, 1978; Last & Greenberg, 1980), and conspicuous accumulation of laminin was found in the lung interstitium of pulmonary fibrosis based on an immunofluorescence study (Singer *et al.*, 1986). In our results, increased laminin P1 levels in serum were also demonstrated in patients with pulmonary fibrosis.

Figure 5 Change in serum laminin P1 and NSE levels in responders of small cell lung cancer before and after combination chemotherapy. These two parameters significantly decreased after the response.

Figure 6 Course of serum laminin P1, NSE, and CEA levels in a limited-disease patient with small cell lung cancer. Laminin P1 and NSE levels fell after combination chemotherapy. With tumour recurrence, laminin P1, NSE, and CEA levels increased. A partial response was induced with salvage chemotherapy with reduction in laminin P1 and NSE levels but CEA level showed a further increase.

In the present study, we attempted to determine whether increased levels of circulating laminin correlate with the presence of distal metastasis or the extent of the disease. However, no significant difference in the level could be found between the patient groups in clinical stages of SCLC. SCLC is a rapidly proliferating neoplasm with a marked tendency toward early metastatic spread. It has been shown that laminin enhances metastases formation of tumour cells (Terranova *et al.*, 1983; Barsky *et al.*, 1984; Hunt, 1989). However, the value of serum laminin P1 for indicator of distal metastasis in SCLC is disappointing.

We also investigated the possibility of the clinical usefulness of laminin P1 as a biomarker in bronchogenic carcinoma in comparison with CEA and NSE. Some tumour marker levels often increase in inflammatory disease. However, in our results, none of the patients with respiratory infection showed a serum laminin P1 level exceeding the cut-off value. A preliminary study has demonstrated that serum laminin P1 level exceeds the upper normal limit in about 50% of tumour patients (Brocks *et al.*, 1986). However, in the present study, the positivity rates of laminin P1 were low in NSCLC (11.5%). The positivity rate and diagnostic accuracy in SCLC were 58.9% and 70.4%, respectively. In view of the sufficient diagnostic accuracy and sensitivity observed in patients with SCLC, serum laminin P1 may be a useful tumour marker in differential diagnosis of a suspected SCLC. It has been shown that serum NSE is a highly specific marker for SCLC (Johnson *et al.*, 1984; Splinter *et al.*, 1987). In our results, the positivity rate of NSE in SCLC was 88.2%, while that for CEA in SCLC was 35.3%. Thus, laminin P1 assay in SCLC offers no superiority in sensitivity over NSE assay, but is superior to CEA.

The present study showed no significant correlation among the levels of serum laminin P1, NSE, and CEA in SCLC. Furthermore, in two SCLC patients with negative NSE, one had positive laminin P1 level. Therefore, these results imply the possibility of a high degree of complementarity for a combined tumour marker analysis.

Serial laminin P1 determination in SCLC was also performed to investigate the potential usefulness of chemotherapeutic monitoring in comparison with NSE. The serum laminin P1 level correlated to therapeutic response, showing a decrease with remission and an increase with recurrence. The results indicate that laminin P1 as well as NSE assay may be useful for therapy monitoring.

We conclude that the sufficient sensitivity to SCLC and specificity of laminin P1 warrants its clinical application as one of the biomarkers in SCLC. However, serum laminin P1 will not help in assessing the occurrence of distal metastasis. We could not verify any superiority of laminin P1 over NSE assay as a marker for SCLC.

### References

BARSKY, S.H., RAO, C.N., WILLIAMS, J.E. & LIOTTA, L.A. (1984). Laminin molecular domains which alter metastasis in a murine model. *J. Clin. Invest.*, **74**, 843.

BIEGLMAYER, C., FEIKS, A. & RUDELSTORFER, R. (1986). Laminin in pregnancy. *Gynecol. Obstet. Invest.*, **22**, 7.

BROCKS, D.G., STRECKER, H., NEUBAUER, H.P. & TIMPL, R. (1986). Radioimmunoassay of laminin in serum and its application to cancer patients. *Clin. Chem.*, **32**, 787.

GERSTMEIER, H., GABRIELLI, A., MEURER, M. & 3 others (1988). Levels of type IV collagen and laminin fragments in serum from patients with progressive systemic sclerosis. *J. Rheumatol.*, **15**, 969.

GREENBERG, D.B., REISER, K.M. & LAST, J.A. (1978). Correlation of biochemical and morphologic manifestations of acute pulmonary fibrosis in rats administered paraquat. *Chest*, **74**, 421.

HÖGEMANN, B., VOSS, B., ALTENWERTH, F.J. & 3 others (1986). Concentrations of 7S collagen and laminin P1 in sera of patients with diabetes mellitus. *Klin. Wochenschr.*, **64**, 382.

HUNT, G. (1989). The role of laminin in cancer invasion and metastasis. *Exp. Cell Biol.*, **57**, 165.

JAPAN LUNG CANCER SOCIETY (1987). *General Rule for Clinical and Pathological Record of Lung Cancer*, 3rd Edition, p. 17, Kanehara: Tokyo.

JOHNSON, D.H., MARANGOS, P.J., FORBES, J.T. & 4 others (1984). Potential utility of serum neuron-specific enolase levels in small cell carcinoma of the lung. *Cancer Res.*, **44**, 5409.

LAST, J.A. & GREENBERG, D.B. (1980). Ozone-induced alterations in collagen metabolism of rat lungs. II. Long-term exposures. *Toxicol. Appl. Pharmacol.*, **55**, 108.

NOUCHI, T., WORNER, T.M., SATO, S. & LIEBER, C.S. (1987). Serum procollagen type 3 N-terminal peptides and laminin P1 peptide in alcoholic liver disease. *Alcohol Clin. Exp. Res.*, **11**, 287.

PIETSCHMANN, P., SCHERNTHANER, G., SCHNACK, C.H. & GAUBE, S. (1988). Serum concentration of laminin P1 in diabetics with advanced nephropathy. *J. Clin. Pathol.*, **41**, 929.

RISTELI, J., ROHDE, H. & TIMPLE, R. (1981). Sensitive radioimmunoassays for 7S collagen and laminin: application to serum and tissue studies of basement membranes. *Anal. Biochem.*, **113**, 372.

ROBERT, P., CHAMPIGNEULLE, B., KREHER, I. & 5 others (1989). Evaluation of fibrosis in the Disse space in noncirrhotic alcoholic liver disease. *Alcohol Clin. Exp. Res.*, **13**, 176.

ROHDE, H., WICK, G. & TIMPLE, R. (1979). Immunochemical characterization of the basement membrane glycoprotein laminin. *Eur. J. Biochem.*, **102**, 195.

ROHDE, H., BÄCHINGER, H.P. & TIMPLE, R. (1980). Characterization of pepsin fragments of laminin in a tumor basement membrane: evidence for the existence of related proteins. *Physiol. Chem.*, **361**, 1651.

SALO, T., LIOTTA, L.A. & TRYGGVASON, K. (1983). Purification and characterization of a murine basement membrane collagen-degrading enzyme secreted by metastatic tumor cells. *J. Biol. Chem.*, **258**, 3058.

SCARPA, S., MORSTYN, G., CARNEY, D.N., MODESTI, A. & TRICHE, T.J. (1988). Small cell lung cancer cell lines: pure and variant types can be distinguished by their extracellular matrix synthesis. *Eur. Resp. J.*, **1**, 639.

SINGER, I.I., KAWKA, D.W., MCNALLY, S.M. & 3 others (1986). Extensive laminin and basement membrane accumulation occurs at the onset of bleomycin-induced rodent pulmonary fibrosis. *Am. J. Pathol.*, **125**, 258.

SNIDER, G.L., HAYES, J.A. & KORTHY, A.L. (1978). Chronic interstitial pulmonary fibrosis produced in hamsters by endotracheal bleomycin. *Am. Rev. Resp. Dis.*, **117**, 1099.

SPLINTER, T.A.W., COOPER, E.H., KHO, G.S., OOSTEROM, R. & PEAKE, M.D. (1987). Neuronspecific enolase as a guide to the treatment of small cell lung cancer. *Eur. J. Cancer Clin. Oncol.*, **23**, 171.

STARCHER, B.C., KUHN, C. & OVERTON, J.E. (1978). Increased elastin and a collagen content in the lungs of hamsters receiving an intratracheal injection of bleomycin. *Am. Rev. Resp. Dis.*, **117**, 299.

TERRANOVA, V.P., LIOTTA, L.A., RUSSO, R.G. & MARTIN, G.R. (1982). Role of laminin in the attachment and metastasis of murine tumor cells. *Cancer Res.*, **42**, 2265.

TERRANOVA, V.P., RAO, C.N., KALEBIC, T., MARGULIES, I.M. & LIOTTA, L.A. (1983). Laminin receptor on human breast carcinoma cells. *Proc. Natl Acad. Sci.*, **80**, 444.

TRYGGVASON, K., HOYHTYA, M. & SALO, T. (1987). Proteolytic degradation of extracellular matrix in tumor invasion. *Biochim. Biophys. Acta*, **907**, 191.

WURZ, H. & CROMBACH, G. (1988). Radioimmunoassay of laminin P1 in body fluids of pregnant women, patients with gynecological cancer and controls. *Tumour Biol.*, **9**, 37.

ZUCKER, S., TURPEENIEMI-HUJANEN, T., WIEMAN, J.M. & LYSIK, R.M. (1988). Characterization of a connective tissue degrading metalloproteinase from human small cell lung cancer cells. *Clin. Expl. Metast.*, **6**, 363.